# High-Resolution Urban Air Quality Mapping for Multiple Pollutants Based on Dense Monitoring Data and Machine Learning

**DOI:** 10.3390/ijerph19138005

**Published:** 2022-06-29

**Authors:** Rong Guo, Ying Qi, Bu Zhao, Ziyu Pei, Fei Wen, Shun Wu, Qiang Zhang

**Affiliations:** 1 Department of Computer Science and Engineering, Northwest Normal University, Lanzhou 730070, China; louisguo626@gmail.com (R.G.); qiying@nwnu.edu.cn (Y.Q.); yuzipe@163.com (Z.P.); 2 School for Environment and Sustainability, University of Michigan, Ann Arbor, MI 48109, USA; zhaobu@umich.edu; 3 Gansu Academy of Eco-Environmental Sciences, Lanzhou 730070, China; wenfei258@126.com; 4 Sichuan Meteorological Service Centre, Chengdu 610072, China; wushunws6@163.com

**Keywords:** air quality mapping, high-resolution, micro monitoring stations, LCS network, machine learning

## Abstract

Spatially explicit urban air quality information is important for urban fine-management and public life. However, existing air quality measurement methods still have some limitations on spatial coverage and system stability. A micro station is an emerging monitoring system with multiple sensors, which can be deployed to provide dense air quality monitoring data. Here, we proposed a method for urban air quality mapping at high-resolution for multiple pollutants. By using the dense air quality monitoring data from 448 micro stations in Lanzhou city, we developed a decision tree model to infer the distribution of citywide air quality at a 500 m × 500 m × 1 h resolution, with a coefficient of determination (R^2^) value of 0.740 for PM_2.5_, 0.754 for CO and 0.716 for SO_2_. Meanwhile, we also show that the deployment density of the monitoring stations can have a significant impact on the air quality inference results. Our method is able to show both short-term and long-term distribution of multiple important pollutants in the city, which demonstrates the potential and feasibility of dense monitoring data combined with advanced data science methods to support urban atmospheric environment fine-management, policy making, and public health studies.

## 1. Introduction

Urban air pollution seriously affects public health in both developed and developing countries [1,2,3]. It is worth noting that more than 50% of the world’s population lives in urban areas where most of the air pollution-related health impact occurs [4].

Because of the severe urban air pollution, there is a growing demand for air quality monitoring services, which aim to understand the real-time air quality of any area in the city (e.g., street, community, school), as well as their spatial–temporal variations at a higher resolution [5]. Such information could greatly help public decision making (such as whether to take exercise outdoors) and urban fine-management and policy making (such as performing controls for the area that often produce severe air pollution) to reduce public health and capital loss. However, inferring the citywide air quality at both a high spatial and temporal resolution can be extremely challenging. First, the standard air quality monitoring stations are limited worldwide due to their high cost and space limitation. Even in affluent regions, air quality monitors are also sparse (i.e., there are 18 continuous regulatory monitors in the New York metropolitan area [6] and 35 in Beijing, China [7]), which provide only limited coverage of air quality monitoring. Second, the air pollutants’ distribution can vary greatly at both small spatial and temporal scales due to complex physical–chemical transformations and multiple emission sources, especially in populous urban areas [5,8]. Third, urban air pollution is a complex dynamic system, which not only has a strong spatial dependency with adjacent areas [9], but also can be affected by a variety of factors (e.g., meteorological, urban structures).

Currently, a variety of methods have been used to infer urban air quality, but still with various limitations. The classical dispersion models and chemical transport models are in most cases a function of meteorology, receptor locations, traffic volumes, and emission factors. These models can offer high fidelity but tend to be computationally expensive and need empirical assumptions and parameters [10,11,12]. Spatial interpolation methods are based on the air quality reports from nearby monitoring stations, which are usually employed by public websites releasing AQIs. However, their inference accuracy is often not guaranteed due to air quality varying across locations non-linearly [13]. Land-use regression can provide air quality estimations with a high spatial resolution but lack sufficient temporal resolution. Meanwhile, it highly relies on the availability of updated local land-use data [14,15,16]. Some studies use satellite remote sensing data to estimate air quality [17,18], but they are usually spatially coarse (1−10 km resolution) [19] and easily affected by cloudy weather and water/snow glint reflectance [20,21].

In recent years, many studies have utilized machine learning methods and low-cost sensor (LCS) technology for urban air quality inference modeling, and it has been proved to have a better performance [5,22]. However, we note that existing studies still needs further improvement. First, most studies based on such as land-use regression or satellite remote sensing lack a sufficient temporal resolution, which can only model daily variations in urban air quality [19,23,24]. In turn, urban air pollution showed obvious differences over continuous hours [5,8]; thus, it is necessary to understand the hourly variations in air quality for public daily decision-making and urban fine-management. Second, regarding spatial resolution, many studies inferred air quality at coarse resolutions (10 km) [23,25,26,27], with only a few exceptions at 1 km [28,29]. However, in multicenter health studies, there are dense residents in many urban areas where air pollution exposures may vary greatly within a 10-km^2^ or smaller grid cells. Third, the LCS sensors have higher spatial–temporal measurement resolution [5,30], but existing LCS-based methods still have some limitations. The portable monitoring devices are not necessarily accurate due to cost and volume limitations, and often focus on a specific area rather than the whole city [14]. The vehicles equipped with sensors cannot guarantee the monitoring time (less observation at night) and are easily affected by human factors (forgetting to open) or operating environments (the wind in driving) [19,31]. Therefore, it is necessary to explore a method to steadily infer the variations in urban air quality at both a high spatial and temporal resolution.

In this paper, we propose a method to address the challenges of high-resolution urban air quality mapping by exploring the potential of combining dense LCS networks and machine learning techniques. Specifically, we collected air quality data from 448 micro monitoring stations (micro station) in Lanzhou City. Then, we developed a decision tree model to infer the citywide air quality at a 500 m × 500 m × 1 h resolution by using these dense monitoring data and external data (meteorological and land-use data). The results show that our method can accurately infer the distribution of and variation in multiple important pollutants at ahigh spatio-temporal resolution, which demonstrates the potential and feasibility of dense monitoring data for urban air quality mapping, and provide support for urban atmospheric environment fine-management, policy making, and public health studies.

## 2. Materials and Methods

### 2.1. Study Area and Micro Stations Distribution

**Figure 1:**
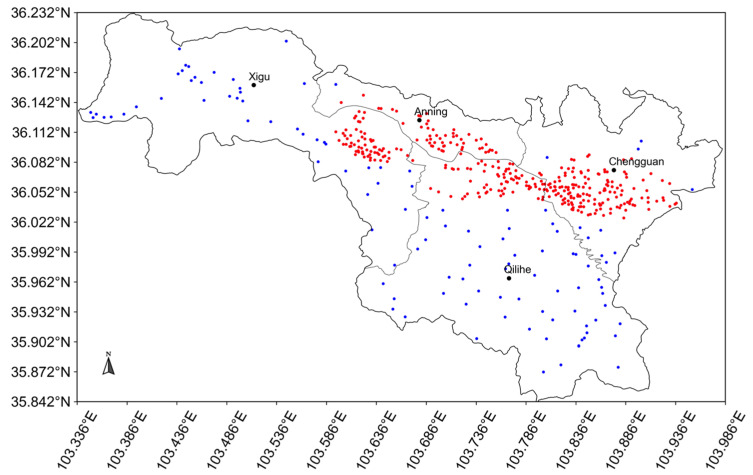
Study area and the distribution of micro stations in Lanzhou City, China, where the red dots represent the stations in the core area.

Lanzhou is the capital of Gansu Province, China, which covers about 1073 km^2^ with four administrative districts. The Xigu District is the core industrial area, Anning District is the science and education center, and Qilihe District and Chengguan District are the business areas. As a center for the petrochemical industry and heavy industry and also an important transportation hub, it suffers from severe air pollution. Therefore, a dense air quality monitoring network with 448 micro stations (about 0.4 stations per square kilometer) has been established here to assist in air pollution control. Most of these stations are located in the core area with a high density (335 stations in 184 km^2^, about 2 stations per square kilometer) (Figure 1). The dense network can monitor the hourly concentrations of both particulate matters (PM_2.5_, PM_10_) and gaseous pollutants (CO, O_3_, NO_2_, and SO_2_) (Supplementary Material Table S1). Figure S1 shows the station distribution number of the micro stations in each administrative district.

### 2.2. Data Collection

#### 2.2.1. Air Quality Data

In this study, we focused on urban air quality in winter, when severe air pollution is more likely to occur and vary greatly (Figure S2). A one-phase air quality data collection campaign was conducted from 11 October 2021 to 19 February 2022 in Lanzhou City. The air quality data comes from 448 micro stations, including real-time air pollutant concentrations, collection timestamp, and collection location (longitude, latitude, and street). A total of 1,339,055 h of air quality monitoring data were obtained after removing erroneous data, and the data-missing rate was 2.3% in the time series. Figure S2 shows the regional average of the air pollutant concentrations during the study period. All micro stations are managed by the Department of Ecology and Environment of Gansu Province, China. They are factory calibrated and regularly maintained to ensure the readings have acceptable precision. More details about the parameters and performance of the monitoring equipment are presented in the Supplementary Material.

#### 2.2.2. Meteorological and Land-Use Data

We also collected meteorological conditions data during the study period. The meteorological data comes from 51 streets in Lanzhou city, including weather, temperature, relative humidity, wind direction, wind level, collection timestamp, and collection location (longitude, latitude, and street). Finally, a total of 118,773 h of meteorological data were obtained. Our study uses the land-use data of Lanzhou city at a 30 m spatial resolution; there are 6 first-level types, including cultivated land, forest land, grassland, water area, construction land, unused land, and 25 s-level types, including forest land, shrubland, sparse forest land, other forest land, and high, medium, and low coverage grassland.

### 2.3. City Grid

First, the geographical coordinate system WGS2000 was used for the geographical registration of the entire study area, and the city vector shape was extracted. We divided the study area into 4139 grids based on the distribution density of the micro stations, and each grid size was 500 m × 500 m. Next, all micro stations, as well as the air quality monitoring data, were assigned to the corresponding grids based on their location (latitude and longitude). The pollutant concentrations of each grid area were from the observed value of its corresponding micro station. For grids that contain multiple stations, we took the average value of the multiple stations and assigned it to these grids. Finally, for all grids in the study area, including the blank grids without assigned micro stations, the spatially adjacent data were searched and allocated to the grids based on the spatial distance between each grid and adjacent units. We obtained high-resolution data for all grids in the study area: (1) Location: longitude and latitude; (2) Air quality data: air quality monitoring data from the nearest 10 micro stations at the same hour (i.e., spatial neighbors); (3) Meteorological data: hourly weather, temperature, relative humidity, wind direction, and wind force data from the nearest street; (4) Land-use data: the size of each of the 25 land-use types in each grid. These data were used in the next step as the input variables of each grid for developing the air quality inference model for the entire study area.

### 2.4. Air Quality Inference Model

#### 2.4.1. Model Construction

For urban air quality, the air pollutant concentrations of a given grid is spatially correlated to its adjacent units. For example, the air quality of a location is likely to be bad if the air quality of its adjacent areas is bad. In addition, external factors, such as meteorological conditions, can affect regional pollutant transport and local pollutant deposition. In order to capture the potential impact of multiple factors on urban air quality, we developed a machine learning model based on Extreme Gradient Boosting (XGBoost) [32] to infer the air quality of a given grid by using multiple spatially adjacent data related to the grid.

**Figure 2:**
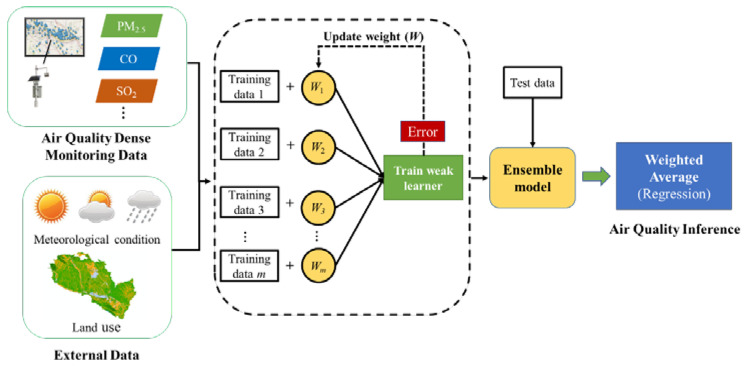
Study framework.

The XGBoost algorithm was improved based on the gradient boosted decision tree (GBDT), which offered additional functions (e.g., column sampling and shrinkage) to avoid overfitting and enhance the model predictability [32]. By introducing the regularization item to measure the complexity of the tree model in the objective function, XGBoost can reduce the risk of model overfitting. The decision tree was used as the basic learner of XGBoost, and the weight of the learner was updated by the error gained from each iteration. Finally, the learners with different weights formed an ensemble model, and the prediction was generated by the weighted average. In general, XGBoost has better accuracy due to its additional training process, and takes less time to build the model. In our study, the nonlinear spatial correlation of air pollution in adjacent areas, heterogeneous external data, and the advantages of XGBoost being fast and accurate were integrated into a universal study framework for urban air quality inference. As shown in Figure 2, our goal is that the framework can derive hourly citywide air quality inference as soon as updated data are available.

#### 2.4.2. Variable Selection and Model Hyperparameters

A variety of methods are used to further construct the inference model. We selected the needed predictors by the feature importance of the XGBoost algorithm, which is a backward elimination procedure (i.e., gradually removing the variables with the lowest importance). The model hyperparameters are optimized by a parameter search method called GridSearchCV with 5-fold cross-validation, choosing the hyperparameters set that minimizes RMSE. The search range of different hyperparameters is shown in Table 1. The experiments were mainly run on a computing server, and the model was built using python (hardware information and software versions are shown in Table S2).

#### 2.4.3. Evaluation Metric

In our study, three metrics were used to evaluate the air quality infer performance, where $y_{i}$ is the true value, ${\hat{y}}_{i}$ is the inference value, and ${\bar{y}}_{i}$ is the sample mean.

Root mean squared error (RMSE):

(1) $$\mathrm{RMSE}=\sqrt{\frac{\sum_{i=1}^{n}{\left(y_{i}-{\hat{y}}_{i}\right)}^{2}}{n}}$$

Coefficient of determination (R^2^):

(2) $$R^{2}=1-\frac{\sum_{i=1}^{n}{\left(y_{i}-{\hat{y}}_{i}\right)}^{2}}{\sum_{i=1}^{n}{\left(y_{i}-\bar{y}\right)}^{2}}$$

Pearson correlation coefficient (COR):

(3) $$\mathrm{COR}=\frac{COV\left({\hat{y}}_{i},\;y_{i}\right)}{\sqrt{Var\left[{\hat{y}}_{i}\right]}Var\left[y_{i}\right]}$$

## 3. Results

### 3.1. Data Analysis

#### 3.1.1. Spatio-Temporal Characteristics for Urban Air Pollutants

**Figure 3:**
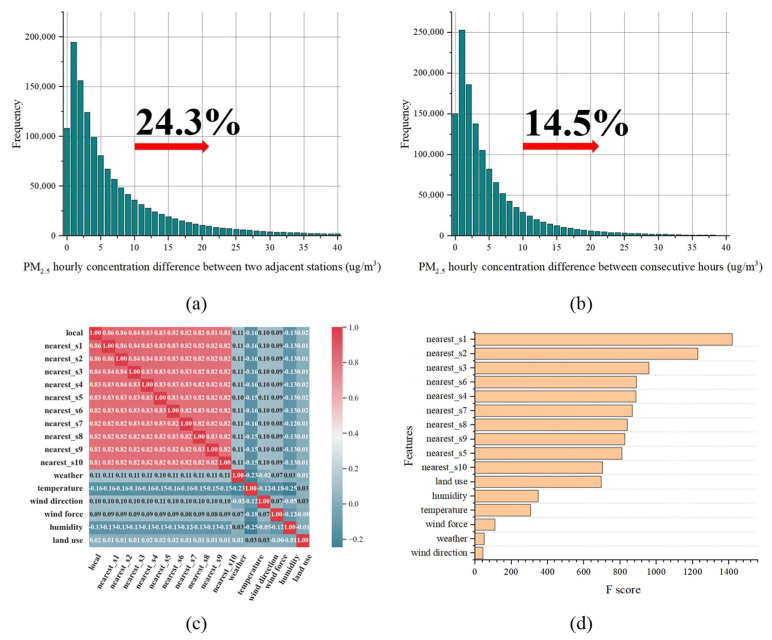
(**a**) PM_2.5_ hourly concentration differences between two adjacent stations at the same hour; (**b**) PM_2.5_ hourly concentration differences for the same stations between two consecutive hours; (**c**) Spearman correlation between PM_2.5_ pollution and the predictors; (**d**) feature importance of different predictors for PM_2.5_ pollution inference.

Taking PM_2.5_ as an example, the highest level of concentration appeared on 15 February 2022, 9:00 p.m. (129.3 micrograms per cubic meter (μg/m^3^)) and the lowest level appeared on 12 October 2021, 8:00 a.m. (17.9 μg/m^3^). For spatial distribution, the highest level of the PM_2.5_ concentration average was observed at a thermal power plant in Xigu District (68.2 μg/m^3^) and the lower level was obtained at a village in the suburbs of Chengguan District (42.7 μg/m^3^). Figure 3a shows the PM_2.5_ concentration differences between two adjacent stations at the same hour during the study period, with about 24.3% of the data having differences higher than 10 micrograms per cubic meter (μg/m^3^). Similarly, with about 14.5% of the data having differences over 10 μg/m^3^ for the same stations between two consecutive hours. Several studies have shown that a 10 μg/m^3^ increase in PM_2.5_ pollution can significantly increase the all-cause mortality as well as respiratory and cardiovascular disease hospitalizations [33,34,35,36]. These results show that urban PM_2.5_ pollution has a relatively significant variation at both small temporal and spatial scales. This may be the result of a complex urban structure, human activities, and the uneven distribution of various emission sources. In addition, it also shows the effectiveness of micro stations to capture the significant spatial heterogeneity of urban air pollutants.

#### 3.1.2. Correlation and Feature Importance

First, Spearman correlation analysis was conducted on PM_2.5_ pollution and Figure 3c shows the correlation between the PM_2.5_ data and other spatially adjacent data. It can be seen that the closer stations have more significant impacts on local PM_2.5_ pollution, which follows the First Law of Geography [37]; i.e., *“Everything is related to everything else, but near things are more related than distant things”*. Meanwhile, the external data are also correlated with PM_2.5_ pollution. Then, we used the XGBoost algorithm to calculate the feature importance of different predictors for PM_2.5_ pollution inference. The result is shown in Figure 3d; overall, the pollution data from the nearest two micro stations have the highest importance for PM_2.5_ inference performance, which is consistent with the result in the Spearman correlation analysis. In meteorological conditions, both relative humidity and temperature are of high importance to PM_2.5_ inference. For the impact of temperature on model inference, this may be due to the winter heating activities that vary with temperature. This phenomenon is consistent with Mateusz Zaręba and Tomasz Danek’s study in Krakow, Poland [38]. They proved that temperature has a direct and important impact on the PM concentration in winter/early spring months. Relative humidity also shows obvious impact on PM_2.5_ inference, which may be related to its fluctuation [39]. Significantly, the wind direction had the lowest F-score for PM_2.5_ inference, which may be closely related to the unobvious wind direction variations in Lanzhou city. Among the meteorological data collected, the northeasterly wind accounts for about 63.6%, and the non-sustained wind accounts for about 19.7%. Similarly, for wind force, about 36% of the data were of the same wind force, which may be the reason for its relatively low impact on PM_2.5_ inferences.

### 3.2. Performance on Air Quality Inference

One common challenge faced by urban air quality inference is the lack of sufficient monitors to provide a spatially distributed benchmark as the ground truth. In our study area, there are 448 micro stations located in various regions, which enable us to develop and validate the air quality inference model based on their monitoring data. Specifically, we first used the data from grids assigned with micro stations for ground truthing, to train and test the air quality inference model. Then, the tested model was used to estimate the air quality of all grids in the study area.

We developed and tested various methods for making air quality inference.

**Support Vector Regression (SVR)**: SVR is expected to find an optimal fitting line so that all data points can be as close as possible to this line, thus making a prediction. The SVR model in our study was developed based on the “sklearn” package in Python.

**k-Nearest Neighbor (KNN)**: The core of the KNN algorithm is to find *k* nearest neighbors of a given sample in the feature space and assign the attributes’ average of these neighbors to the sample for prediction. KNN and SVR are both classical regression algorithms, which are taken as the baselines to compare the performance improvement of existing popular algorithms in air quality inference tasks. The KNN model in our study was developed based on the “sklearn” package in Python, and the number of neighbors was selected as three.

**Deep Neural Network (DNN)**: DNN is a kind of artificial neural network, which receives a variety of predictors as inputs from the input layer, and by training the neurons in hidden layers, the final air quality inference results are produced in the output layer. With a large number of training samples, DNN often has excellent performance, but may also need more computing resources. We used DNN as one of the baselines to evaluate its time-cost and accuracy in the air quality inference task. The DNN model in our study was developed based on the “Keras” package in Python, and there is one input layer (64 neurons) and two hidden layers (32 and 16 neurons).

**Random Forest (RF)**: RF is composed of multiple classification or regression trees. The input predictors are randomly split into each tree using a bootstrap method, and the data in each tree are employed to train the prediction model. It tends to have a lower risk of model overfitting and we chose it as one of the baselines. The RF model in our study was developed based on the “sklearn” package in Python, and n_estimators = 100, min_samples_split = 2 were used. 

**XGBoost**: As one of the most popular algorithms in regression tasks, its tree structure often achieves the better performance in both time cost and accuracy. Meanwhile, the feature importance from XGBoost is helpful for the understanding of the inferred results. For the XGBoost model, it was developed based on the “xgboost” package in Python, and n_estimators = 100, learning_rate = 0.08, max_depth = 7, min_child_weight = 3, colsample_bytree = 1, and subsample = 1 were used.

The air pollutant concentrations from the adjacent micro stations at the same hour, meteorological data, and land-use data were taken as input variables. For categorical variables (e.g., weather, land-use data), we used the one-hot encoding to transform them into feature vectors. In total, 70% of the data were used as the training set and the rest, 30%, as the test set. Finally, we tested the inference performance of three important air pollutants (PM_2.5_, CO, and SO_2_) in winter, and all the pollutants’ data were analyzed following the procedures in Section 3.1. The performance of each method on the test set is shown in Table 2.

Overall, the XGBoost model performs the best on all three metrics, and DNN, Random Forest, and XGBoost have a similar performance. Significantly, the XGBoost model has the fastest model computing speed, with 12 s on the training set and 0.09 s on the test set. In contrast, the Random Forest model took 1 min 31 s on the training set and 3.12 s on the test set. The DNN model took the longest time, 24 min 55 s, on the training set, and 3.8 s on the test set. The parallelized tree construction may be one of the reasons that the XGBoost model has the lowest time cost compared to other models, and it can greatly facilitate the model deployment in real-world practice. The XGBoost model has good inference performance for hourly concentrations of three pollutants, which proves that our model results are stable and robust for inferring multiple pollutants in the city. Furthermore, Table 3 shows the comparison of the impact of different predictors on the model performance, which shows that more micro stations, as well as external data, are beneficial for air quality inference performance.

**Figure 4:**
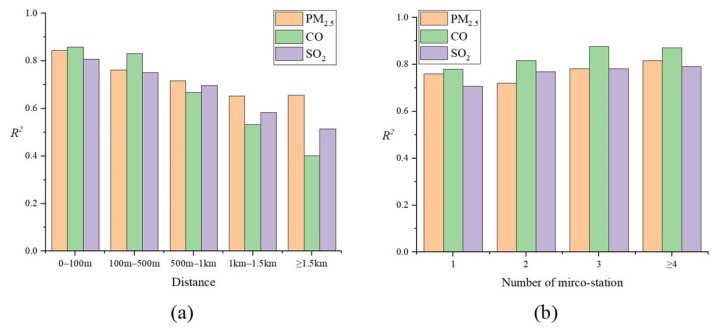
The impact of dense monitoring data on model performance: (**a**) by different distances between each grid and its nearest micro stations; (**b**) by different numbers of micro station.

Then, we divided the grid data in the test set into five categories based on the distance between each grid and its nearest micro stations. It helps to understand how the dense monitoring data affect the model performance for different grids. As shown in Figure 4a, overall, the closer monitoring points can further improve model performance, and the good performance comes from the grids with micro stations within a radius of 500 m (500 m-grids). We then further explored the impact of the number of micro stations on model performance for the 500 m-grids (Figure 4b). Similarly, more micro stations perform better for air quality inference in general. These results indicate that there are very fine-grained spatial variations in urban air quality, and it is important to consider these variations to improve the accuracy of air quality inference.

### 3.3. Mapping Citywide Air Quality at a High Resolution

#### 3.3.1. Continuous Variations in the Short Term

**Figure 5:**
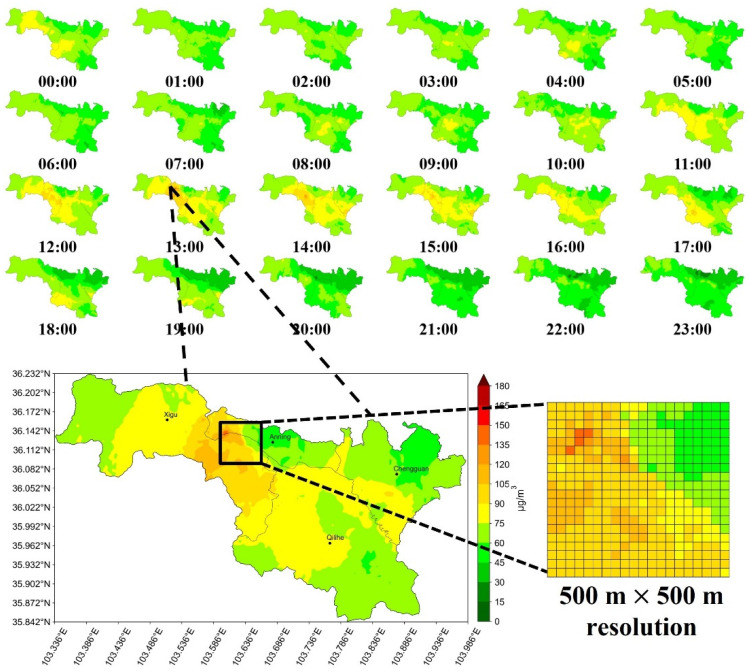
Hourly PM_2.5_ concentration distribution on a typical weekday (8 February 2022) in Lanzhou, as inferred by our model.

Figure 5 shows the spatial and temporal distribution of PM_2.5_ pollution measured continuously for 24 h (8 February 2022, a typical weekday) in Lanzhou, inferred by the best-performing XGBoost model. According to the hourly inference results, the citywide PM_2.5_ concentration remained at a relatively low level between 01:00 and 07:00, which is consistent with the fact of suspension of industrial and human activities during midnight. The PM_2.5_ concentration in the core area began to rise from 11:00, and at 13:00, we can clearly identify a pollution hotspot in Xigu district and its dissipation within four hours. This pollution hotspot inferred by our model is also consistent with a maximum observation of 136 μg/m^3^ and an average of 96.46 μg/m^3^ reported by micro stations in the area. Our results are able to show the citywide air quality at a 500 m × 500 m × 1 h resolution, which is useful for assessing air pollution exposure at multiple locations within a 500 m × 500 m grid. Several studies, as well as our results, have shown that urban air quality has obvious variations at both small temporal and spatial scales [5,8,19]. Therefore, it is of great significance for urban fine-management and health research to infer urban air quality at the smallest possible scale.

#### 3.3.2. Long-Term Distribution

**Figure 6:**
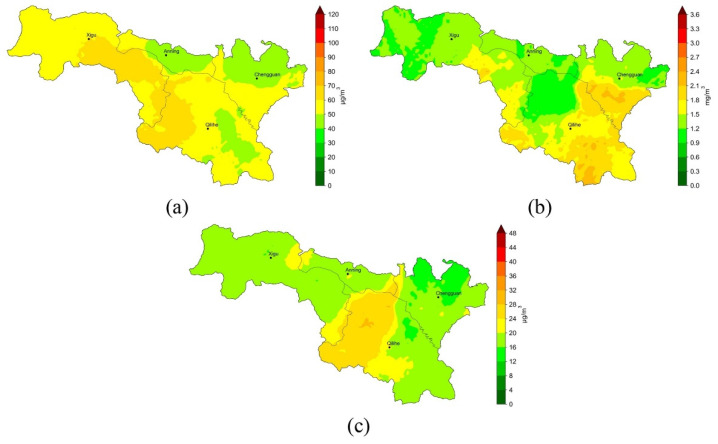
Average concentrations inferred by our model during 8–12 February 2022 in Lanzhou city: (**a**) PM_2.5_; (**b**) CO; (**c**) SO_2._

Figure 6 depicts the long-term average distribution of PM_2.5_, CO, and SO_2_ pollution in the study area inferred by our model. Obviously, there was a great difference in the long-term distribution of air pollutants in different areas. Xigu district has a higher PM_2.5_ level, which may be closely related to the intensive industrial activities. In contrast, the Anning district and the northern suburbs of the Chengguan district have a lower PM_2.5_ level. For CO pollution, the Chengguan district and the southern Qilihe district has higher pollution levels. This may be due to intensive vehicle emissions in these areas. The SO_2_ pollution is mainly concentrated in the west of the Qilihe district, and hourly concentration observation in this area also shows the same distribution trend. This suggests that SO_2_ pollution is likely to be closely related to specific local emission sources. The long-term inferred results from our method can provide important information for urban management and policy making.

#### 3.3.3. More Monitors Are Always Better?

**Figure 7:**
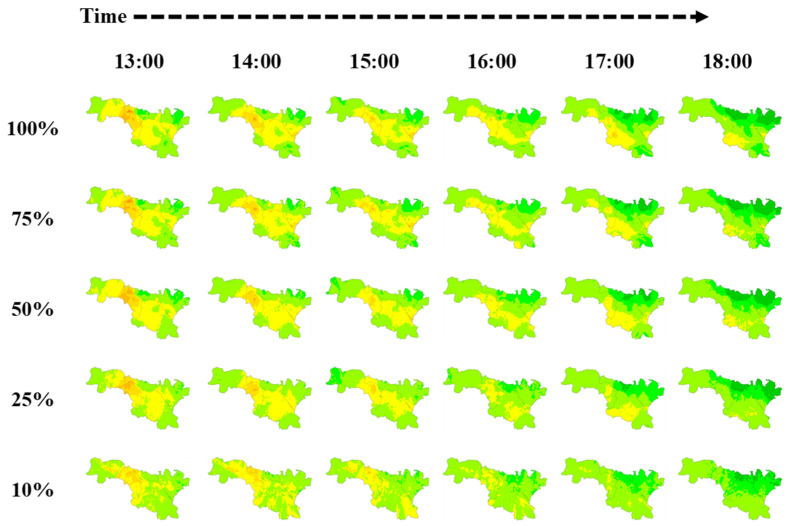
Citywide PM_2.5_ pollution mapping results on 8 February 2022 by different station densities.

Figure 4 shows the impact of micro station distribution on inference performance for a given grid. However, it is not clear how many deployed stations are sufficient for a particular city to achieve acceptable performance. Therefore, we further explored the impact of different monitoring network densities on the citywide air quality mapping results. Specifically, we randomly selected 10%, 25%, 50%, and 75% from 448 micro stations to map citywide PM_2.5_ pollution based on our model. As shown in Figure 7, with the number of stations decreasing, the inference performance for PM_2.5_ pollution gradually decreases. Specifically, it is difficult to accurately infer PM_2.5_ hotspots and variations when the number of stations is 10% (less than 0.05 micro stations per square kilometer, and still more than the number of standard stations deployed in most cities). This is mainly because without the dense monitoring data, the model inputs for each grid are largely similar without significant variations. This suggests that more monitoring data are always beneficial, and there is a trade-off between the monitoring costs and the inference accuracy, as fewer micro stations can still provide rough inferences about the air quality variations. Therefore, for those areas in which deploying LCS networks is considered, they can decide on the monitoring network density based on their budget and the severity of the pollution.

## 4. Discussion

In recent years, there has never been a bigger need for user-focused urban air quality monitoring services in support of safe, healthy, and resilient cities [5,40]. Especially for industrial cities such as Lanzhou, air quality monitoring is particularly important to guide urban management, public decision making, and health studies. In fact, in our results, the Xigu district is more prone to severe PM_2.5_ pollution than other regions, which may be closely related to its role as an industrial cluster. This pattern of pollution distribution has been confirmed by other studies [41,42]. There are significant differences in the long-term distribution of multiple pollutants, which may be due to the uneven distribution of emission sources in the city (e.g., industrial emissions are mainly from Xigu district). For Lanzhou, as a city in northern China, the frequent heating activities in winter have a significant influence on air quality variations. Another study in Krakow also demonstrated this phenomenon [38]. Meanwhile, in our results, land-use information has an obvious importance for air quality inference, which is consistent with the findings of a study on the relationship between land use and air quality in Lanzhou city [43].

Our results show the obvious variations in urban air quality at a finer temporal and spatial resolution, which may be closely related to the complex urban structure, human activities, or multiple emission sources [8,44]. The high-resolution air quality inferences may be useful for multicenter health studies with highly dense urban populations. A variety of methods have been used for urban air quality inference, but still lack sufficient spatio-temporal coverage, resolution, and sustainability to meet social needs [5,14,19,45]. The lack of sufficient monitoring data and stable monitoring equipment may be the reason for this progress being hindered. In addition, compared with the dense monitoring network in our study, it is difficult to continuously model spatial correlation of air quality between adjacent areas due to the sparse distribution of air quality monitoring stations. Thus, they may ignore a lot of spatial information, leading to inaccurate inference. Our method can complement atmospheric studies to provide both a high temporal and spatial resolution of multiple important pollutants in the city, which cannot be differentiated in previous studies.

For urban air quality mapping, more community efforts are needed. Our method can be applied to other similar cities or regions for air quality inference studies. They can fine-tune our model to infer the air quality in the target city by using their monitoring data. This is especially important for cities that do not yet have sufficient air quality monitoring measures. In addition, we further show the impact of station deployment density on air quality infer performance, which can provide valuable references for air pollution management in other cities.

Urban air quality is a complex dynamic system, which is affected by many factors. In this study, the feature importance from the XGboost algorithm is crucial for us to efficiently select predictors that are beneficial for model inference. In future work, more potential effects and factors (e.g., other pollutants, traffic, and points of interest (POI)) should be further considered, which is helpful to improve the accuracy and reliability of the air quality inferences. Furthermore, it is also worth studying the influence relationship between the different pollutants by their inferred results.

## 5. Conclusions

In this paper, we proposed a method for high-resolution urban air quality mapping based on dense monitoring data and machine learning techniques. We applied the method in Lanzhou City using the air quality monitoring data from 448 micro stations in winter. The results show that the XGBoost model we developed can accurately infer the spatio-temporal distribution of and variations in urban air pollutants at a 500 m × 500 m × 1 h resolution, with an R^2^ value of 0.740 for PM_2.5_, 0.754 for CO, and 0.716 for SO_2_. The inferred short-term variations in PM_2.5_ missions clearly identify pollution hotspots and their transitions throughout the day. Meanwhile, the inferred long-term distribution of multiple pollutants is significantly different, which may be due to the uneven distribution of emission sources in the city. We also compared the impact of the distance and density of station deployment on air quality inference performance. The results indicate that more stations are always beneficial as they provide more spatial information. However, one also needs to consider the balance between monitoring costs and inference accuracy in real urban management.

## Figures and Tables

**Table 1:** Hyperparameters search range in the GridSearchCV.

Hyperparameter	Range	Interval
n_estimators	100~500	100
learning_rate	0.05~0.1	0.01
max_depth	3~10	1
min_child_weight	1~6	1
colsample_bytree	0.7~1	0.1
Subsample	0.7~1	0.1

**Table 2:** Model performance comparison.

	PM_2.5_			CO			SO_2_		
Methods	RMSE	R^2^	COR	RMSE	R^2^	COR	RMSE	R^2^	COR
KNN	12.710	0.653	0.814	0.524	0.659	0.816	6.426	0.618	0.794
SVR	11.666	0.708	0.851	0.518	0.668	0.858	6.135	0.652	0.844
DNN	11.171	0.732	0.860	0.452	0.747	0.865	5.607	0.709	0.842
Random Forest	11.188	0.731	0.856	0.455	0.743	0.862	5.645	0.705	0.840
XGBoost	10.999	0.740	0.861	0.445	0.754	0.869	5.537	0.716	0.846

**Table 3:** The impact of different predictors on the XGBoost model performance.

	PM_2.5_			CO			SO_2_		
Predictors	RMSE	R^2^	COR	RMSE	R^2^	COR	RMSE	R^2^	COR
1 station	12.820	0.647	0.806	0.567	0.602	0.784	6.806	0.571	0.763
3 stations	11.414	0.721	0.849	0.469	0.727	0.853	5.879	0.680	0.825
5 stations	11.183	0.732	0.856	0.458	0.740	0.861	5.649	0.705	0.840
7 stations	11.093	0.736	0.858	0.452	0.747	0.864	5.569	0.713	0.844
10 stations	11.055	0.738	0.859	0.449	0.750	0.866	5.550	0.715	0.846
10 *s* + *m* ^1^	11.045	0.738	0.859	0.449	0.750	0.866	5.545	0.715	0.846
10 *s* + *l* ^2^	11.021	0.739	0.860	0.447	0.753	0.868	5.538	0.716	0.846
10 *s* + *m* + *l*	10.999	0.740	0.861	0.445	0.754	0.869	5.537	0.716	0.846

^1^ Meteorological data; ^2^ Land-use data.

## Data Availability

Not applicable.

## Supplementary Materials

The following supporting information can be downloaded at: https://www.mdpi.com/article/10.3390/ijerph19138005/s1, Figure S1: The distribution number of micro stations in each administrative district; Figure S2: The regional average of air pollutant concentrations during study period; Table S1: Air pollutants monitoring data from micro station No. 12395 on 25 October 2021; Table S2: Experiment environment; Table S3: Device parameters for air quality monitoring; Table S4: Device performance indicators of particulate matters monitoring; Table S5: Device performance indicators of gaseous pollutants monitoring.
