# Supplementary material for: High-Resolution Urban Air Quality Mapping for Multiple Pollutants Based on Dense Monitoring Data and Machine Learning
Source: Int J Environ Res Public Health. 2022 Jun 29;19(13):8005. doi: 10.3390/ijerph19138005 (PMC9265361; doi:10.3390/ijerph19138005)
Supplement: Supplementary file 1 [file ijerph-19-08005-s001.zip › ijerph-1722118-supplementary.pdf]

Supplementary Material for

High-Resolution Urban Air Quality Mapping for Multiple Pollutants based on Dense Monitoring Data and Machine Learning

Rong Guo [1], Ying Qi [1], Bu Zhao [2], Ziyu Pei [1], Fei Wen [3], Shun Wu [4], Qiang Zhang [1,*]

[1] *Department of Computer Science and Engineering, Northwest Normal University, Lanzhou, Gansu Province, 730070, China*

[2] *School for Environment and Sustainability, University of Michigan, Ann Arbor, MI, 48109, USA*

[3] *Gansu Academy of Eco-environmental Sciences, Lanzhou, Gansu Province, 730070, China*

[4] *Sichuan Meteorological Service Centre, Chengdu, Sichuan Province, 610072, China*

* *Corresponding author's e-mail: zhangq@nwnu.edu.cn*

This PDF file includes:

Table S1-S5
Figure S1-S2

Supplementary Tables:

Table S1. Air pollutants monitoring data from micro-station No.12395 on 2021/10/25.

Time	$PM_{2.5}$(μg/m^3)	PM_{10}(μg/m^3)	SO_2(μg/m^3)	NO_2(μg/m^3)	CO(mg/m^3)	O_3(μg/m^3)
14:00	30	45	8	44	0.42	69
15:00	31	48	8	56	0.495	53
16:00	25	35	8	52	0.442	44
17:00	28	43	9	45	0.398	51

Table S2. Experiment environment.

OS	Ubuntu 20.04.3 LTS
Memory	32GB
CPU	Intel(R) Xeon(R) Silver 4210 CPU @ 2.20GHz
GPU	NVIDIA RTX 3090
Python version	3.8.2
Keras version	2.7.0

Table S3. Device parameters for air quality monitoring.

Version	XHAQSN-812
Monitoring parameters	$PM_{2.5}$、PM_{10}、TSP、CO、O_3、SO_2、NO_2、NO、TVOC
Dimension	240×230×170 mm
Weight	3kg
Power supply	urban electricity system, solar energy
Rated power	5W
Communication mode	4G/3G/2G
Working environment	T(-20～55)°C、RH(0%～95%) No condensation

Table S4. Device performance indicators of particulate matters monitoring.

Monitoring parameters	$PM_{2.5}$、PM_{10}、TSP
Measurement range	(0 ~ 2000, 30000) μg/m^3
Minimum resolution	1μg/m^3

Table S5. Device performance indicators of gaseous pollutants monitoring.

Monitoring parameters	SO_2	NO_2	O_3	CO	NO	TVOC
Measurement range	(0~500) nmol/mol	(0~500) nmol/mol	(0~500) nmol/mol	(0~500) nmol/mol	(0~50) μmol/mol	(0~20) or (0~50) μmol/mol
Error	±10% FS	±10% FS	±10% FS	±10% FS	±10% FS	±5% FS (5~50 μmol/mol) ±10% FS (0~5 μmol/mol)
Repeatability	5%	5%	5%	5%	5%	1%

Supplementary Figures:

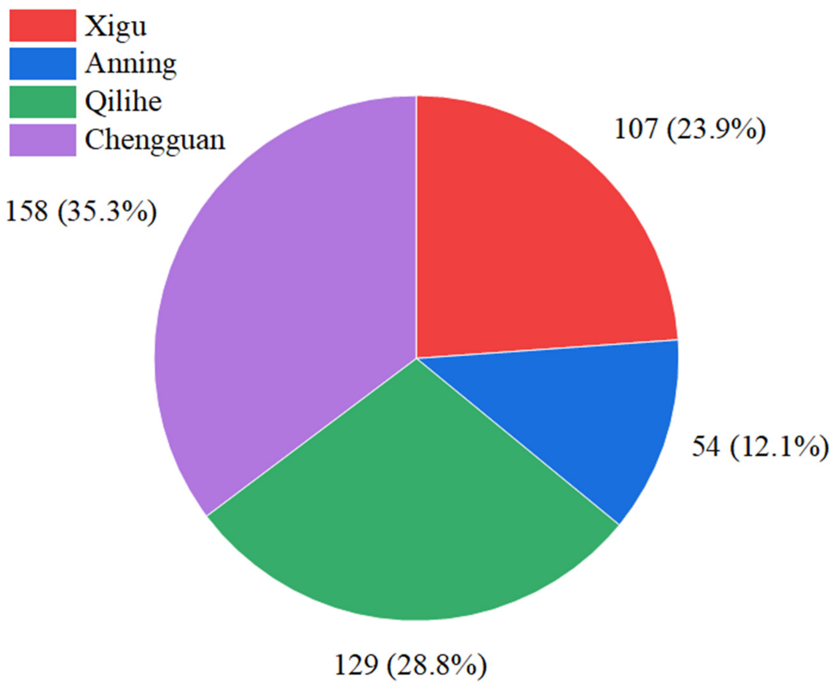

Figure S1. The distribution number of micro-stations in each administrative district.

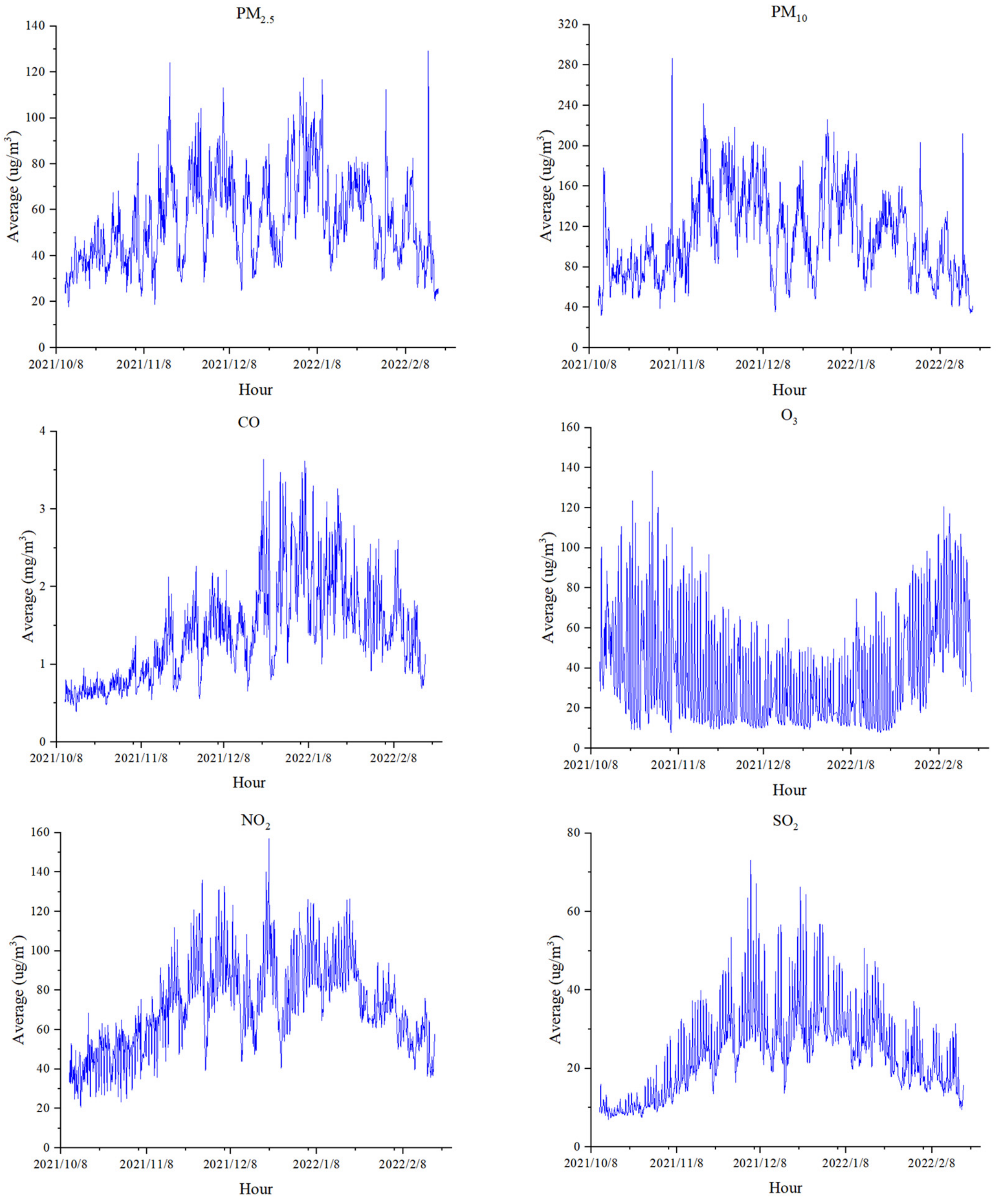

Figure S2. The regional average of air pollutant concentrations during study period.
